# Spatial Distribution of Stem Cell-Like Keratinocytes in Dissected Compound Hair Follicles of the Dog

**DOI:** 10.1371/journal.pone.0146937

**Published:** 2016-01-20

**Authors:** Dominique J. Wiener, Marcus G. Doherr, Eliane J. Müller, Monika M. Welle

**Affiliations:** 1 Institute of Animal Pathology, Vetsuisse Faculty, University of Bern, Bern, Switzerland; 2 Dermfocus, Vetsuisse Faculty, Inselspital, Bern University Hospital, Bern, Switzerland; 3 Institute of Veterinary Epidemiology and Biostatistics, Department of Veterinary Medicine, Free University of Berlin, Berlin, Germany; 4 Department of Dermatology, Inselspital, Bern University Hospital, Bern, Switzerland; Faculty of Animal Sciences and Food Engineering, University of São Paulo, BRAZIL

## Abstract

Hair cycle disturbances are common in dogs and comparable to some alopecic disorders in humans. A normal hair cycle is maintained by follicular stem cells which are predominately found in an area known as the bulge. Due to similar morphological characteristics of the bulge area in humans and dogs, the shared particularity of compound hair follicles as well as similarities in follicular biomarker expression, the dog is a promising model to study human hair cycle and stem cell disorders. To gain insight into the spatial distribution of follicular keratinocytes with stem cell potential in canine compound follicles, we microdissected hair follicles in anagen and telogen from skin samples of freshly euthanized dogs. The keratinocytes isolated from different locations were investigated for their colony forming efficiency, growth and differentiation potential as well as clonal growth. Our results indicate that i) compound and single hair follicles exhibit a comparable spatial distribution pattern with respect to cells with high growth potential and stem cell-like characteristics, ii) the lower isthmus (comprising the bulge) harbors most cells with high growth potential in both, the anagen and the telogen hair cycle stage, iii) unlike in other species, colonies with highest growth potential are rather small with an irregular perimeter and iv) the keratinocytes derived from the bulbar region exhibit characteristics of actively dividing transit amplifying cells. Our results now provide the basis to conduct comparative studies of normal dogs and those with hair cycle disorders with the possibility to extend relevant findings to human patients.

## Introduction

In the dog, like in humans and mice, hair follicles (HFs) self-renew and undergo recurrent phases of controlled growth (anagen), regression of the lower portion of the follicle through apoptosis of keratinocytes (catagen), and relative quiescence (telogen) [1]. After a variable time in telogen, a new HF forms in the anagen phase which occurs, as best characterized in the mouse, through activation of stem cells (SCs) in response to inductive signals from the dermal papilla, the surrounding mesenchyme and the SC niche [2]. If the tightly controlled growth phases of the hair cycle (HC) are disturbed, non-inflammatory alopecia is a frequent and common problem in humans and dogs. The spatial allocation of HF SCs is therefore crucial to allow future investigations into molecular pathways involved in follicular SCs activation and regulation of the HC.

SCs are “slow-cycling”, relatively undifferentiated cells with high proliferative potential [3–4]. In mouse, human and dog HFs the SC bearing regions, responsible for the cyclical reconstitution of the lower portion of the HF, are considered to be present in the isthmus, which begins at the opening of the sebaceous gland duct and ends at the insertion point of the arrector pili muscle [5–8]. Human and dogs further share species-specific differences compared to the mouse. Within the isthmic structure, the best characterized SC compartment in the mouse is located in the lower half of the isthmus in a morphologically visible trochanter-like extrusion called the bulge [5, 9–10], defined by the attachment site of the arrector pili muscle [11]. In humans and dogs the bulge area has no distinct morphological structure. It is identified in the anagen HC by the attachment of the arrector pili muscle and in the telogen stage due to its location at the lower end of the HF [7, 9, 12]. In the mouse, SCs are also found outside the bulge region. For example, LGR6+ cells were identified in the central isthmus directly above the bulge and are considered as the most primitive epidermal SCs in adult murine HFs [13]. LGR6+ cells can exit their niche during the HC and contribute to the formation of the hair germ. So far little is known about extra-bulge SCs in human and dogs. Another prominent morphological difference compared to the mouse is that humans and dogs possess compound HFs. These consist of an assemblage of 2–15 HFs sharing the same orifice. So far the spatial distribution of SCs in the compounds has not been defined. Besides the afore-mentioned morphological distinctions, differences also exist with respect to biomarker expression in the bulge area of humans and dogs compared to the mouse [10, 14–15].

SCs can be identified by their colony-forming efficiency *in vitro* which reflects the growth potential of cells in a given population. In addition Barrandon and Green defined different colony types derived from human epidermal keratinocytes, namely holoclones, meroclones and paraclones [16]. Holoclones are large cell populations with a smooth perimeter derived from epidermal SCs, as judged by the ability of single cells to be clonally expanded. In contrast to holoclones, meroclones and paraclones are small cell populations with an irregular diameter derived from progenitor and terminal differentiated cells [16]. In a later study Kobayashi et al. investigated the colony forming efficiency of keratinocytes isolated from single canine HFs of the anagen stage [7, 10]. They found that most colonies with holoclone-like morphology and characteristics originated from the isthmus of the HF.

In principle, SC-bearing colonies of human, mouse and canine origin have been reported to be larger than 3mm^2^ [7, 16–17]. However, we recently showed that the majority of colonies with high proliferative potential (as defined by Ki67 expression in all cells of the colony) isolated from microdissected canine HFs had an irregular perimeter, similar to mero- or paraclones described by Barrandon and Green [16], and a diameter smaller than 3mm^2^. Therefore we suggested that colonies with highest growth potential are small colonies with slow cycling cells [8], which is a characteristic of HF SCs *in vivo*.

As outlined above, dogs and humans share many common HF features such as morphological similarities of the bulge area, similarities in hair types, anagen driven HCs (at least for some dog breeds), resemblance in marker expression [10, 18] as well as the occurrence of analogous alopecic disorders. The dog appears therefore to be a very promising model to study human follicular SC biology in non-inflammatory alopecia. This study was therefore designed to gain further insights into the spatial distribution of SCs in canine compound HF and lay the basis to study the pathogenetic mechanisms involved in primary non-inflammatory alopecia.

To reach our goal we investigated the growth potential of keratinocytes derived from compound and single canine HFs in healthy dogs by 1) assessing the colony forming efficiency of cells derived from defined locations of HFs; upper isthmus, lower isthmus and bulbar region, 2) investigating the growth potential compared to premature differentiation of resulting colonies using proliferation and differentiation markers; 3) comparing colony growth during different HC stages (anagen and telogen, respectively) and 4) investigating clonal expansion of canine follicular keratinocytes. In contrast to work done so far on canine follicular SCs, we assessed compound HFs in our study, included HFs in the telogen hair cyle stage and, to localize potential canine SCs more precisely within the HF, subdivided the isthmus further into two compartments (upper and lower isthmus) [7, 10]. Furthermore we assessed the proliferation and differentiation potential of the colonies, which usually is not performed, in terms of specific marker expression.

## Methods

### Skin biopsies

Skin samples were obtained from 29 freshly euthanized dogs of various breeds with no skin abnormalities. All dogs were privately owned pets that were euthanized according to ethical standards of the Canton Bern, Switzerland for reasons unrelated to this study. Samples were taken after euthanasia with the consent of their owners and were stored in Dulbecco’s Modified Eagle Medium (DMEM) (Gibco, Luzern, Switzerland) supplemented with 10% bovine serum and 1% penicillin-streptomycin-fungizone (Amimed, Basel, Switzerland) at 4°C for no more than 16h prior to microdissection.

### Medium

CELLnTEC-09 medium (CnT-09) was purchased from CELLnTEC, Bern, Switzerland and prepared according to the instruction manual. To reduce microbial contamination, 1% penicillin-streptomycin-fungizone (Amimed, Basel, Switzerland) was added.

### Feeder layer

3T3 fibroblasts [19] were seeded at a density of 2x10^5^ cells/ 35mm petri dish on a coverslip (18mmx18mm). After 2hrs cells were adherent to the cover slip and treated with mitomycin c for 2hrs at 37°C. Thereafter the 3T3 cells were maintained in DMEM supplemented with 10% bovine serum and 1% penicillin/streptomycin/fungizone until they were used as feeder cells.

### Microdissection of hair follicles

**Fig 1:**
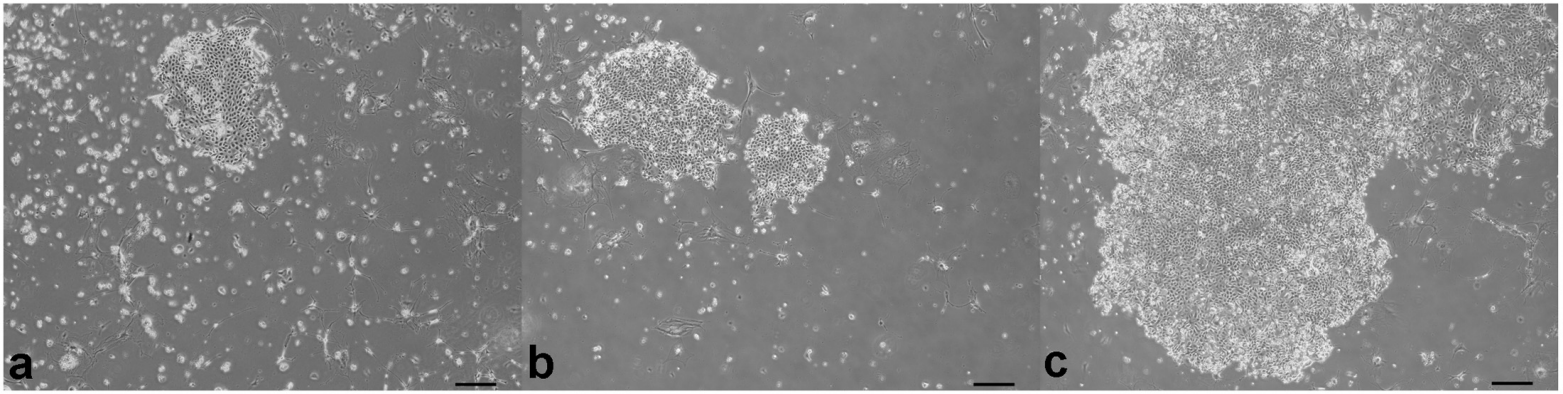
Colony types derived from compound anagen canine hair follicles on a 3T3 feeder layer. a) Colony derived from the upper isthmus. b) Colonies derived from the lower isthmus. c) Colonies derived from the bulbar region. Note confluency of the colonies derived from the bulbar region. Scale bar represents 100μm.

As described previously [8], the biopsy specimens collected in cell culture medium (described above) were placed in PBS. Follicular compounds as well as single HFs were microdissected from the same dogs and utilized for the experiments. The follicular stage was assigned by the length of the hair shaft and the depth of the follicle within the tissue [1, 20]. For this study compound HFs were used where the primary HFs were in the late anagen (anagen VI) or the telogen HC stage. The experiments were performed with single HFs in anagen from 19 dogs and telogen from seven dogs. Furthermore follicular compounds in anagen were microdissected from 11 dogs and in telogen from seven dogs. From each dog two compound and two single follicles (duplicates) were isolated. Follicles were isolated from the surrounding collagen and microdissected as previously described [8]. In the microdissected compound HFs the vast majority of the primary and the secondary HFs were all in the same HC stage. The single HFs and the compounds in anagen were cut above the opening of the sebaceous glands duct and sliced horizontally into three portions, namely upper isthmus, lower isthmus (containing the bulge) and bulbar region (S1 Fig). The telogen single HFs and the compounds were cut horizontally into two fragments, namely upper and lower isthmus (S2 Fig), as the inferior portion is not present in this HC stage. HF fragments were manually collected and treated with trypsin ⁄ EDTA for 1h at 37°C to obtain a single cell suspension of follicular cells. Individualized keratinocytes from each compound were then centrifuged at 1200rpm for 5min and thereafter resuspended in 1ml CnT-09 medium and directly seeded in a petri dish covered with a 3T3 feeder layer, prepared as described above. To avoid substantial cell loss, keratinocytes derived from the individual HF fragments were not counted but seeded directly on the feeder layer to perform the colony forming assays. The medium was changed every third to fourth day. Twelve days after plating, resulting colonies (Fig 1) were evaluated further as described below.

### Immunofluorescence

The colonies growing on the cover slip were stained with Ki67 (proliferation marker (rabbit, clone SP6, 1:100 diluted in 2% FCS-PBS, Cell Marque)) and involucrin (differentiation marker (rabbit, clone Erli, 1:80 diluted in 2% FCS-PBS)) antibodies as described previously [8].

**Fig 2:**
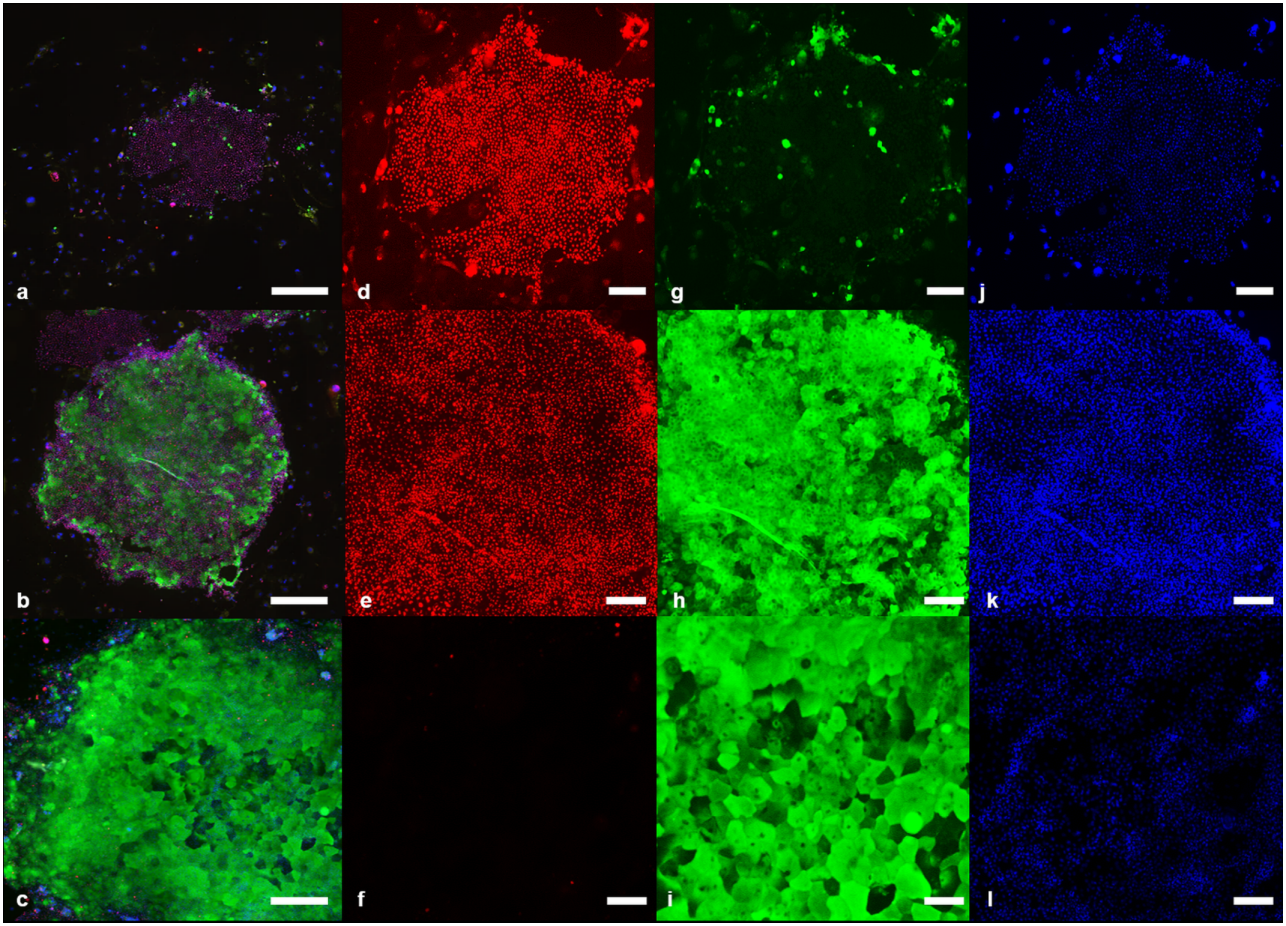
Colony types with three different staining patterns. a) Colony type with mainly proliferative (Ki67+) cells. Note the small size and the irregular shape of the colony. b) Mixed colony type with both proliferative (Ki67+) and terminally differentiated (involucrin+) cells. c) Colony type with mainly terminally differentiated (involucrin+) cells. d-j) Higher magnification of a) depicting Ki67+ cells, involucrin+ cells and DAPI staining, respectively. e-k) Higher magnification of b) depicting Ki67+ cells, involucrin+ cells and DAPI staining, respectively. f-l) Higher magnification of c) depicting Ki67+ cells, involucrin+ cells and DAPI staining, respectively.a-c) Scale bar represents 500μm. d-l)Scale bar represents 200μm. Red: Ki67+ keratinocytes, green: Involucrin+ keratinocytes, blue: Nuclear staining (DAPI).

The stained colonies on the cover slip were photographed with the Zeiss AxioImage fluorescence microscope and evaluated with the AxioVision 4.9.2. software program (Zeiss, Feldbach, Zürich, Switzerland). Colonies ≥400μm in diameter were counted and divided in colonies < 3mm^2^ and > 3mm^2^. The total number of colonies as well as the number of the three resulting colony types (mainly Ki67 positive, mixed Ki67 and involucrin positive and mainly involucrin positive colonies, Fig 2) were counted, evaluated and statistically analyzed as described previously [8].

### Clonal growth of colonies

Clonal expansion of colonies was performed for each location within the telogen (upper isthmus, lower isthmus) and the anagen follicle (upper isthmus, lower isthmus and bulbar region) in five and 12 dogs, respectively. One colony with holoclone-like morphology (>3mm^2^, smooth perimeter) [8] was picked from each location after 12 days with a sterile tooth-pick and incubated in trypsin ⁄ EDTA for 20min at 37°C to get a single cell suspension. Ten single cells were then collected with a pipette. Five of these cells were seeded individually in separate petri dishes which contained a cover slip coated with a 3T3 feeder layer (for immunofluorescence staining) and five single cells were individually seeded in separate petri dishes without cover slip on a 3T3 feeder layer (for ongoing passaging of the colonies after the next 12 days). The remaining harvested single cell suspension was counted and a defined number of cells were seeded in 4 petri dishes coated with a 3T3 feeder layer (10’000, 1’000, 100 and 10 cells per petri dish, respectively). The medium was changed every third to fourth day. After 12 days the colonies in the petri dishes with and without cover slips were fixed in 4% paraformaldehyde and stained with Ki67 and involucrin by immunofluorescence or with rhodamine and counted, respectively. Prior to fixation a single colony (derived from the petri dishes without cover slip seeded with single cell and/or with a defined number of cells) with holoclone-like morphology [16] was picked with a sterile tooth pick and reseeded as described above to evaluate the long-term proliferative capacity of the cells.

### Statistical analyses

The total number of colonies as well as the number of the individual colony types were statistically evaluated for all dogs using the Kruskal-Wallis ANOVA (*P* = 0.05) and rank sum test (*P* < 0.0167), respectively. All statistical analyses were performed using the software package Number Cruncher Statistical System 2008 (NCSS 8, licensed by Jerry L. Hintze, Kaysville, Utah, USA).

## Results

All experiments were performed with single HFs and compound HFs from anagen (19 and 11 dogs, respectively) and telogen (seven dogs) HC stages with comparable results. Shown are results derived from experiments with follicular compounds.

### Number of colonies

Overall, there was a substantial variation in the number of colonies derived from follicular compounds in both the anagen and telogen HC stage between the individual dogs. The total number of colonies ranged from 0 up to 78 in anagen compounds and from 0 to 73 in telogen compounds (data not shown). The majority of the HFs taken in duplicates from the same dog as single and compound HFs yielded a comparable number of colonies. In a few dogs the number of colonies also varied between the duplicates (e.g. duplicates of compound HFs yielded 31 and 68 colonies, respectively, in a dog with anagen HFs and 32 and 58 colonies, respectively, in a dog with telogen HFs).

#### Anagen hair cycle stage

**Fig 3:**
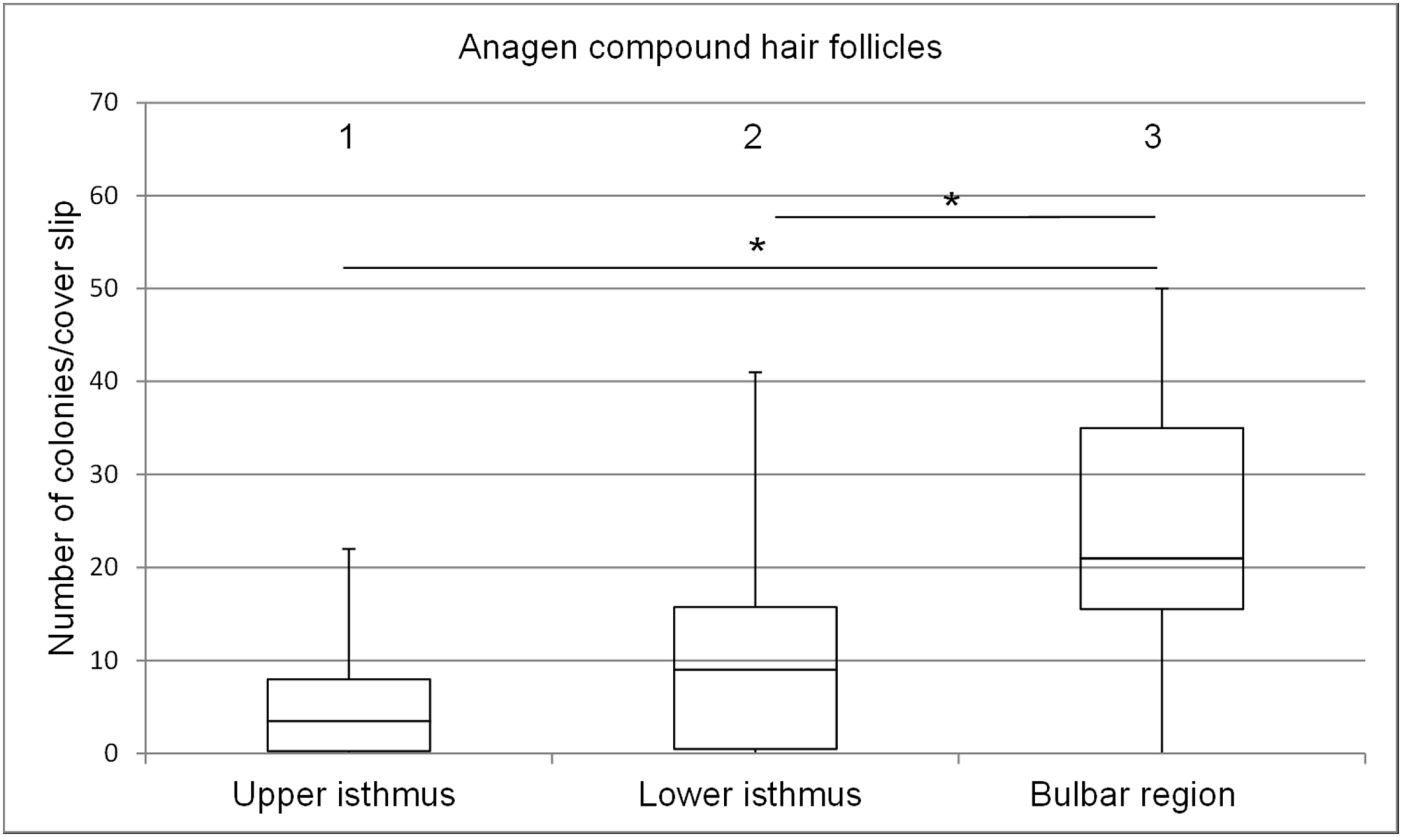
Box plots depicting the range of colony numbers per cover slip growing from keratinocytes of anagen follicular compounds. Colonies derived from the upper isthmus, the lower isthmus and the bulbar region (n = 11). The horizontal line within the box plots indicates the median of the samples. The edges of the box mark the first and third quartiles.

The total number of colonies per cover slip derived from follicular keratinocytes of the bulbar region was significantly higher than from keratinocytes derived from the upper and lower isthmus (Fig 3) with mean numbers of 24.6, 6.0 and 11.0, respectively. The difference between the upper and lower isthmus was not significant (Fig 3, columns 1 and 2). Remarkably, the colonies derived from the bulbar region had the largest diameter and frequently overlapped, however, the border of individual colonies could still be distinguished.

#### Telogen hair cycle stage

**Fig 4:**
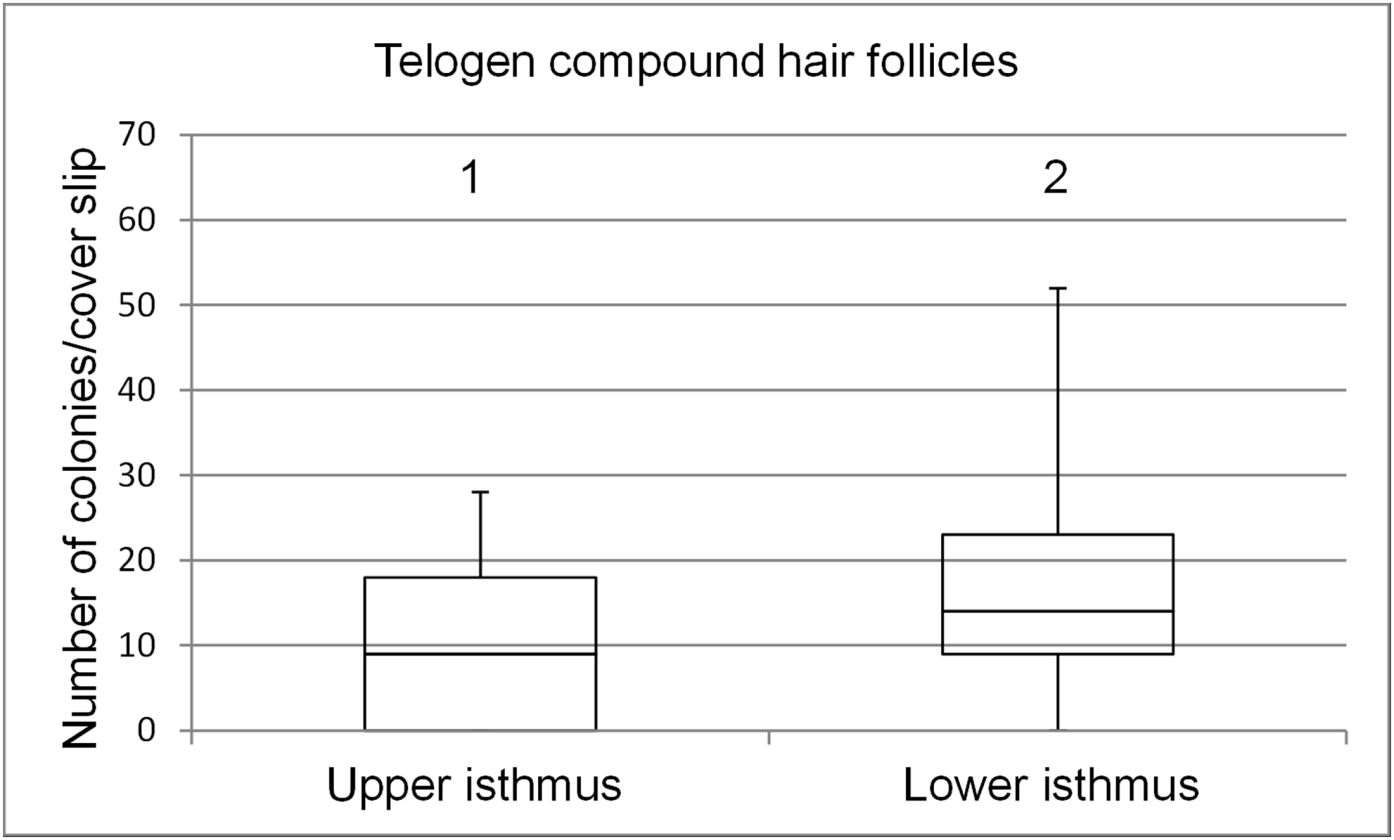
Box plots depicting the range of colony numbers per cover slip growing from keratinocytes of telogen follicular compounds. Colonies derived from the upper isthmus and the lower isthmus (n = 7). The horizontal line within the box plots indicates the median of the samples. The edges of the box mark the first and third quartiles.

Like in the anagen HC stage the number of colonies was higher from keratinocytes derived from the lower isthmus than from the upper isthmus (mean number of colonies 17.1 and 9.1, respectively), however, the difference was not statistically significant (Fig 4, column 1 and 2).

### Growth and differentiation potential

The colonies derived from the different HF fragments of anagen and telogen HC stages were stained with Ki67 and involucrin to distinguish proliferating cells from terminally differentiated cells, respectively. Three types of colonies could be identified, as previously described [8]: Colonies that were either predominantly positive for Ki67 or involucrin and colonies that were positive for Ki67 and involucrin (Fig 2).

#### Anagen hair cycle stage

**Fig 5:**
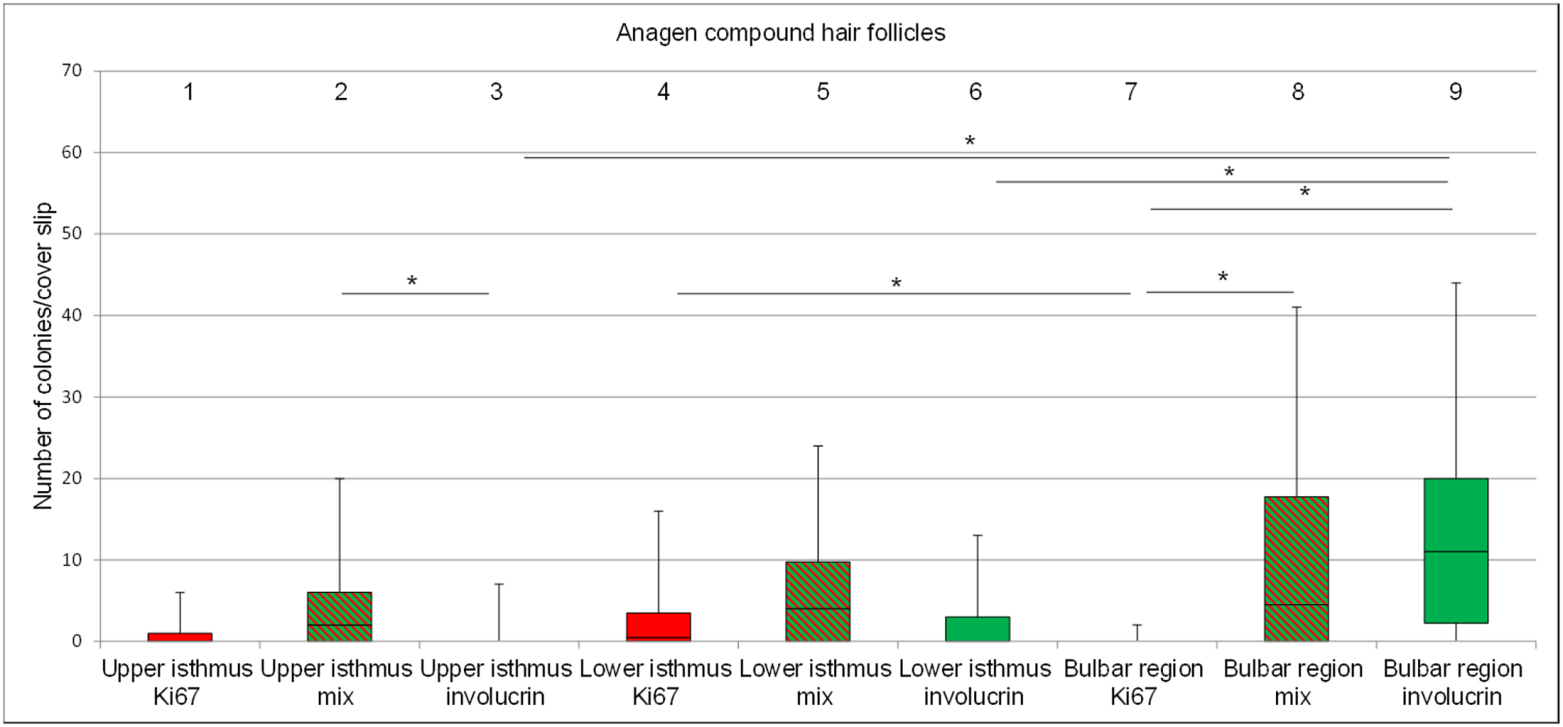
Bar plots representing the percentage of proliferative, mixed type and terminally differentiated colonies per cover slip. Colonies are derived from keratinocytes of the upper isthmus, the lower isthmus and the bulbar region of anagen follicular compounds (n = 11). Red: Ki67+ colonies; green: involucrin+ colonies; red and green stripes: mixed type of colonies. The horizontal line within the box plots indicates the median of the samples. The edges of the box mark the first and third quartiles.

**Fig 6:**
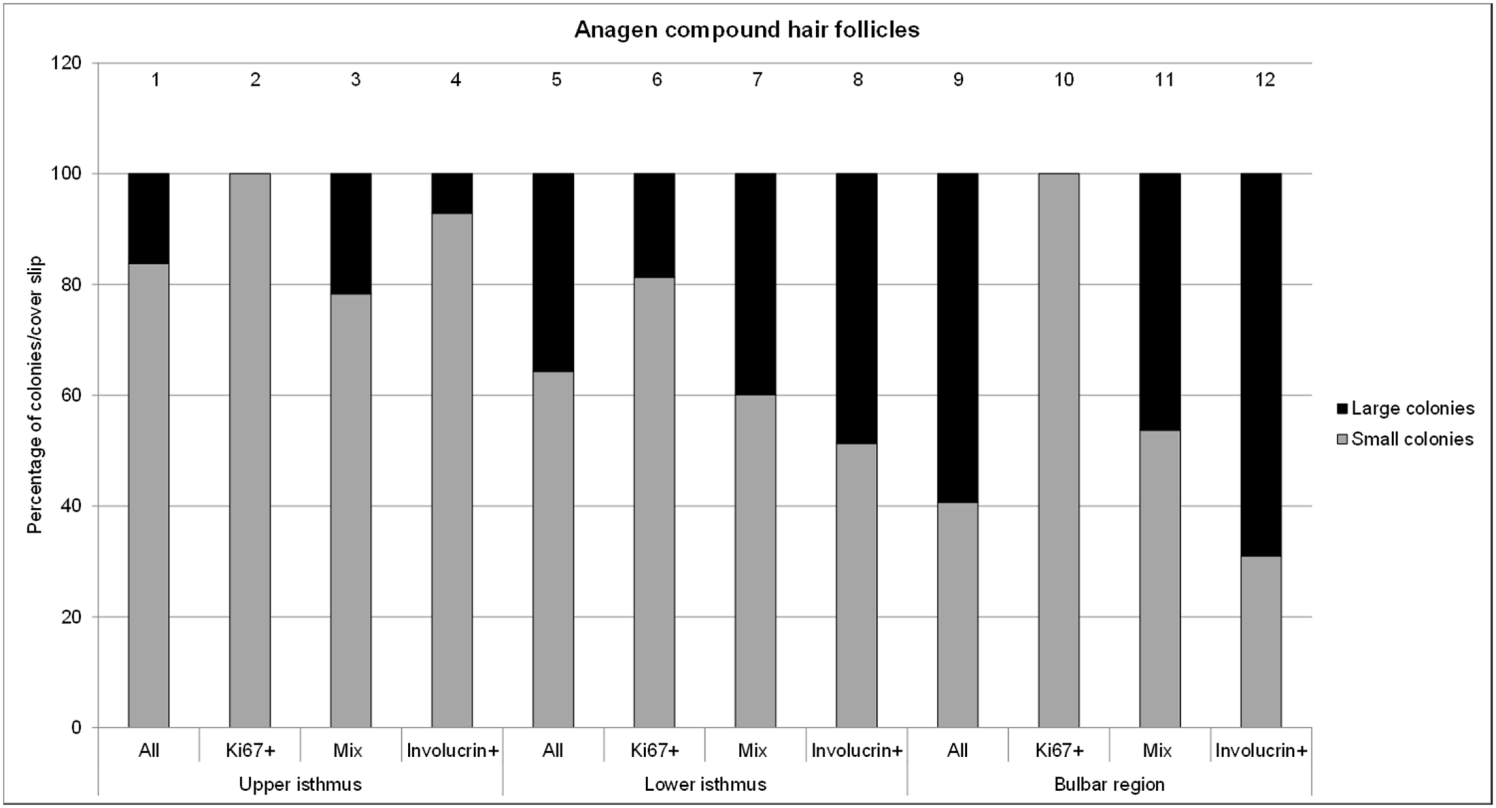
Percentage of small (<3mm^2^) and large (>3mm^2^) colonies derived from the upper isthmus, lower isthmus and the bulbar region (n = 12 dogs). Total percentage of small and large colonies are put together in one column and sum up to 100% (n = 11).

Colonies derived from the bulbar region were significantly more terminally differentiated (involucrin+ colonies) compared to the colonies derived from the upper and lower isthmus, respectively (Fig 5, columns 3, 6 and 9; columns 7, 8 and 9;). On the other hand, significantly more Ki67+ colonies were growing from the lower isthmus than from the bulbar region (Fig 5, columns 4 and 7). Most Ki67+ colonies were present in the lower isthmus containing the bulge (Fig 5, column 4). The majority of the Ki67+ colonies in all HF compartments were smaller than 3mm^2^ (Fig 6, columns 2, 6 and 10) and had a rather irregular perimeter (Fig 2a). Strikingly, almost 60% of the colonies derived from the keratinocytes of the bulbar region were larger than 3mm^2^ (Fig 6, column 12), had also an irregular perimeter (Fig 2c).

#### Telogen hair cycle stage

**Fig 7:**
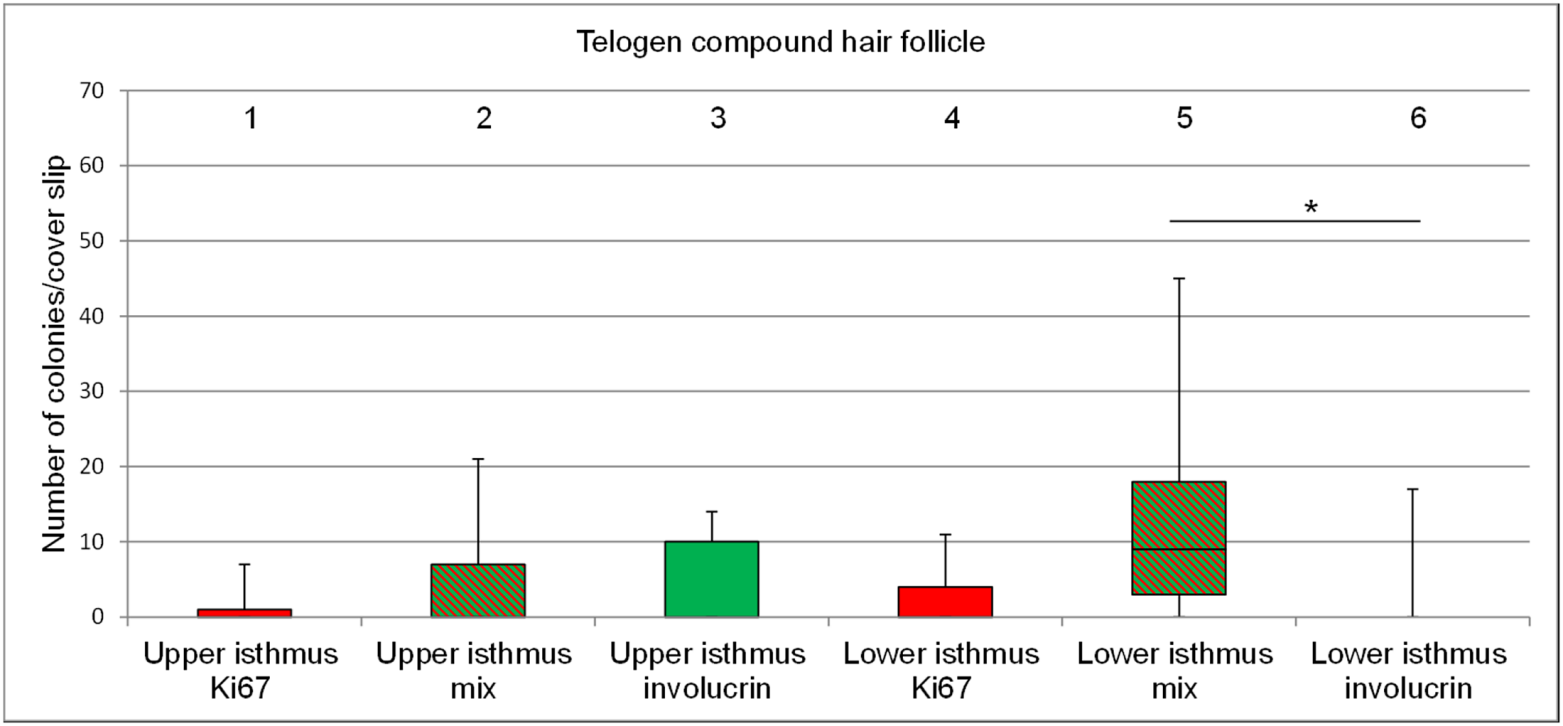
Bar plots representing the percentage of proliferative, mixed type and terminally differentiated colonies per cover slip. Colonies are derived from cells of the upper isthmus and the lower isthmus of telogen follicular compounds (n = 7). Red: Ki67+ colonies; green: involucrin+ colonies; red and green stripes: mixed type of colonies. The horizontal line within the box plots indicates the median of the samples. The edges of the box mark the first and third quartiles.

In contrast to the colonies derived from keratinocytes of the anagen compounds the colonies derived from the telogen compounds had more involucrin positive colonies in the upper isthmus than in the lower isthmus (Fig 7, columns 3 and 6). Most of the proliferative colonies (Ki67+) were present in the lower isthmus (Fig 7, column 4).

**Fig 8:**
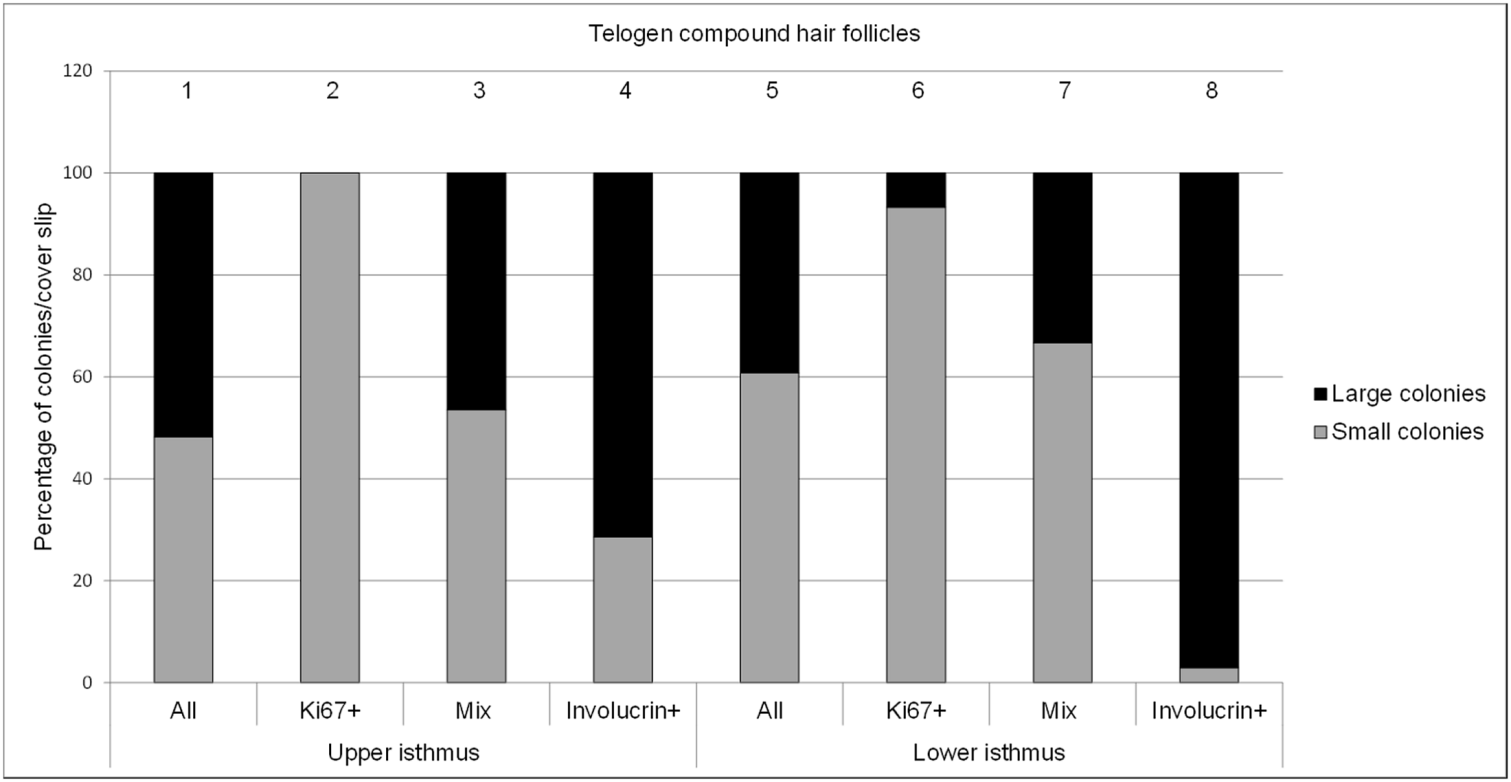
Percentage of small (<3mm^2^) and large (>3mm^2^) colonies derived from the upper isthmus and lower isthmus (n = 7 dogs). Total percentage of small and large colonies are put together in a column and sum up to 100% percent (n = 7).

Like in the anagen follicle the majority of proliferative colonies (Ki67+) were smaller than 3mm^2^ (Fig 8, columns 2 and 6) and the terminally differentiated colonies were larger than 3mm^2^ (Fig 8, column 4 and 8).

### Clonal expansion of follicular keratinocytes and long-term proliferative capacity

#### Clonal growth of follicular keratinocytes

**Anagen hair cycle stage:** Clonal growth of colonies from a single cell isolated from holoclone-like (larger than 3mm^2^ with a smooth perimeter) [16] primary colonies was examined for 12 dogs. Clonal expansion was possible for 6.9%, 15.7% and 8.2% of single cells from the upper isthmus, the lower isthmus and the bulbar region, respectively (data not shown). After passaging of single cells the colonies showed, like the original colony, holoclone-like morphology and were of the mixed type (both Ki67 and involucrin positive).

**Telogen hair cycle stage:** Clonal growth of colonies derived from a single cell was examined for five dogs. In 6.0% and 18% of all seeded single cells derived from the upper isthmus and the lower isthmus, respectively, clonal growth of colonies occurred and colonies were of the mixed type (both Ki67 and involucrin positive).

#### Long-term proliferative capacity

**Anagen hair cycle stage:** As assessed for 12 dogs, the majority of the colonies derived from the upper isthmus could be passaged once (45.4%). Almost a third of the colonies could be passaged twice (27.3%) and 9.1% of the colonies could be passaged three times. 18.2% of the colonies could not be passaged at all. The majority (54.5%) of the colonies derived from the lower isthmus could be passaged twice, 27.3% of the colonies could be passaged once and 18.2% could be passaged three times. The majority (44.4%) of the colonies derived from the bulbar region could be passaged once, 33.3% of the colonies could be passaged twice, 11.1% of the colonies could be passaged three times and 11.1% of the colonies could not be passaged at all. On average, the keratinocytes could be passaged 1.3, 1.9 and 1.8 times when derived from the upper isthmus, the lower isthmus and the bulbar region, respectively.

**Telogen hair cycle stage:** For the five dogs, 25% of the colonies derived from the upper isthmus could be passaged once, twice and four times, respectively. 25% could not be passaged at all. The colonies derived from the lower isthmus could be passaged either once (50%) or four times (50%). On average, the keratinocytes could be passaged 1.75 and 2.5 times when derived from the upper and the lower isthmus, respectively.

## Discussion

The goal of this study was to gain insight into the spatial distribution follicular SC in the canine HF to lay the basis for the elucidation of the pathogenetic mechanisms resulting in primary alopecia in dogs and humans. We defined the relative growth potential of follicular keratinocytes from different locations of the HF and from the main HC stages (anagen and telogen). Furthermore, our interest was in compound HFs, primarily because the compound structure is a special feature, distinguishing the canine and human hair coat from the mouse fur (composed of single HFs only). Compound HFs have not been investigated so far and in canine non-inflammatory alopecia, the secondary HFs of the compound HF are often retained in the kenogen HC stage [21]. Previous studies localized the SC bearing region in the anagen canine HFs to the isthmic region [7]. By subdividing the isthmus in an upper and lower part we can now more accurately locate cells with the highest growth potential to the lower, bulge-containing isthmus in the anagen and telogen stage based on the evaluation of Ki67 expression and clonal expansion of single cells derived from primary follicular colonies. In analogy with mouse and human, we consider the cells with high proliferative potential as SCs [16–17]. Furthermore the assumption that the cells of the Ki-67+ colonies may have SC potential is supported by our yet unpublished results on the expression of SC associated biomarkers [18], which also showed the expression of SC markers in the lower isthmus. However, we are aware that the ultimate proof for the SC potential of these colonies is missing.

### Number of colonies and differentiation/growth potential

In accordance with our findings previous studies showed that K15- and CD34-positive cells (potential canine SC biomarkers) are present mainly in the lower half of the isthmus in telogen [7, 18]. However, in the anagen HC stage CD34- and K15-positivity was found in the outer root sheath of the entire isthmus [7, 18]. As Ki67+ colonies were growing also from keratinocytes derived from the upper isthmus, this agrees with our results and reflects that SCs are present throughout the isthmus with an accumulation in the lower isthmus (containing the bulge). This reflects recent findings in the mouse, where Lgr6+ SC were found above the multipotent bulge [13]. The lowest number of Ki67+ colonies was found in the bulbar region in our studies in agreement with low SC potential of these areas in rodents and humans [22–23]. In contrast, mixed type colonies were distributed in all three locations of the HF, and we suggest that they represent differentiating transit amplifying cells.

In our study the colonies that were mainly Ki67+ were smaller than 3mm^2^ and thus showed no holoclone-like morphology. We assume nevertheless that these colonies are the SC- or early progenitor cell-bearing colonies because of their proliferative capacity. This observation suggests that the growth behaviour of canine SCs and early progenitor cells are different from that of mouse and human. They reflect however the growth behaviour of SCs in these species *in vivo*. The definition of “holoclones” originates from colony-forming human epidermal cells. However previous studies [7, 10] described colonies with holoclone-like morphology in the bulge region of canine HFs and the authors attributed SC properties to these colonies. In agreement with them [7, 10] we also found in our study colonies with holoclone-like morphology derived from the isthmus of canine HFs. However, most of the colonies with holoclone-like morphology were of the mixed type, suggesting (based on their staining pattern) that they are differentiating transit amplifying cells rather than colonies with SC properties.

In our study a significantly higher number of colonies were growing from cells of the bulbar region in comparison to the upper and lower isthmus in the anagen HC stage. In addition these colonies had an irregular perimeter and large diameter bearing large cells. This finding is in contrast to published results from rats and human where only very few colonies could be grown from cells of the bulbar region [22–23]. Furthermore in these experiments the colonies derived from the lower HF region were small with an irregular perimeter identifying them as meroclone-like [16]. In contrast to the findings in humans and rats previous experiments using canine HF keratinocytes are in partial agreement with our results showing the formation of high numbers of colonies with an irregular perimeter derived from cells of the lower part (including the bulbar region) of the canine HF [7]. However, in contrast to our study the colonies presented by Kobayashi et al. were rather small whereas in our experiments, most colonies derived from the bulbar region were larger than 3mm^2^. Despite the large size of the colonies we identified these colonies as terminally differentiated based on their staining properties (involucrin+). Thus, the high number and the size of the colonies growing from bulbar region may reflect the high proliferative potential of matrix cells, which is in agreement with the *in vivo* situation.

The statistical assessment of differences in terms of colony formation and colony subtypes between different locations and different HC stages, respectively, was difficult due to the uneven distribution of the data between the individual dogs and sometimes even between duplicates from the same dog. The striking differences between the individual dogs reflects that not only the intrafollicular microenvironment but also extrafollicular factors such as the molecules derived from the dermis or the intra-dermal adipose tissue, the general physiological status (like the body hormone status, the immune function, neural activities, and age) as well environmental factors influence the HF and SC homeostasis stage [24]. However, it cannot be excluded that some variation may also be the results of the manual microdissection. Despite the uneven distribution of our data we were able to perform a statistical analysis since we investigated HFs from a large number of dogs.

### Clonal expansion and long-term proliferative capacity

Independently of the location of the HF, follicular colony forming cells only underwent a small number of generation doublings. This stands in contrast to the results from Rochat et al., where human follicular colony-forming cells underwent a high number of generations. However, Rochat et al. transferred colonies 7 days after seeding [23]. Previously published results show that after 7 days colonies derived from canine follicular keratinocytes are not yet differentiated and all colonies show more or less the same morphology [8]. In order to differentiate between holoclone- and meroclones/paraclone-like morphology we transferred colonies only after 12 days. This might explain the smaller number of passages in our study. However, the most likely explanation for the low passage number we received may be that we picked single cells from colonies with holoclone-like morphology and not from the smaller colonies. As discussed above, the colonies with the “classical” holoclone morphology generated from microdissected canine HFs were mostly of the mixed type. Therefore, by picking cells from holoclone-like colonies for passaging, we selected a majority of differentiating transit amplifying cells. Unlike us Kobayashi et al. was able to passage canine isthmic keratinocytes on average for almost eight generations [10], whereas in our study colonies could be passaged only 1.3, 1.9 and 1.8 times, respectively, depending on the location where the colonies were derived from. This discrepancy, in addition to the reasons explained above, might be the result of the substantial variation in the colony forming capacity between individual dogs observed here. Furthermore Kobayashi et al. investigated only three dogs whereas we examined colonies from 17 dogs.

Despite the fact that we could not achieve many passages the colonies derived from the lower isthmus could be passaged slightly more often than the colonies derived from other locations (in the anagen as well as in the telogen HFs), indicating that the lower isthmus is the region with the greatest SC potential. This result is in line with previous results in humans [23], where most clonogenic cells were found above the level of the bulb. In both the anagen and the telogen HC stages the clonal growth in lower isthmus which comprises the area of the bulge (15.7% and 18%) was substantially higher than in the upper isthmus (6.9% and 6%, respectively) and the bulbar region (8.2%).

## Conclusions

Taken together our results indicate that in canine compound HFs the cells with the greatest SC potential are located in the lower isthmus in the anagen as well as in the telogen HC stage. Interestingly, we found the colonies with the greatest proliferative potential to be rather small and with an irregular perimeter. Therefore, we suggest that unlike in other species, canine SC-bearing colonies show no “classical” holoclone morphology.

Our findings are important to further investigate molecular aspects of the canine HF SC compartments in health and disease, which might be a promising model for comparative studies with human diseases.

## Supporting Information

S1 Fig Microdissection of compound hair follicles in the anagen hair cycle stage. Hair follicles were microdissected in three fragments: Upper isthmus, lower isthmus and bulbar region. Inf, infundibulum; UpIst, upper isthmus; LoIst, lower isthmus; Bulb, bulbar region.
(TIF)

S2 Fig Microdissection of compound hair follicles in the telogen stage. Hair follicles were microdissected in two fragments: Upper and lower isthmus. Inf, infundibulum; UpIst, upper isthmus; LoIst, lower isthmus.
(TIF)
