# Supplementary figures and images for: Spatial Distribution of Stem Cell-Like Keratinocytes in Dissected Compound Hair Follicles of the Dog
Source: PLoS One. 2016 Jan 20;11(1):e0146937. doi: 10.1371/journal.pone.0146937 (PMC4720375; doi:10.1371/journal.pone.0146937)

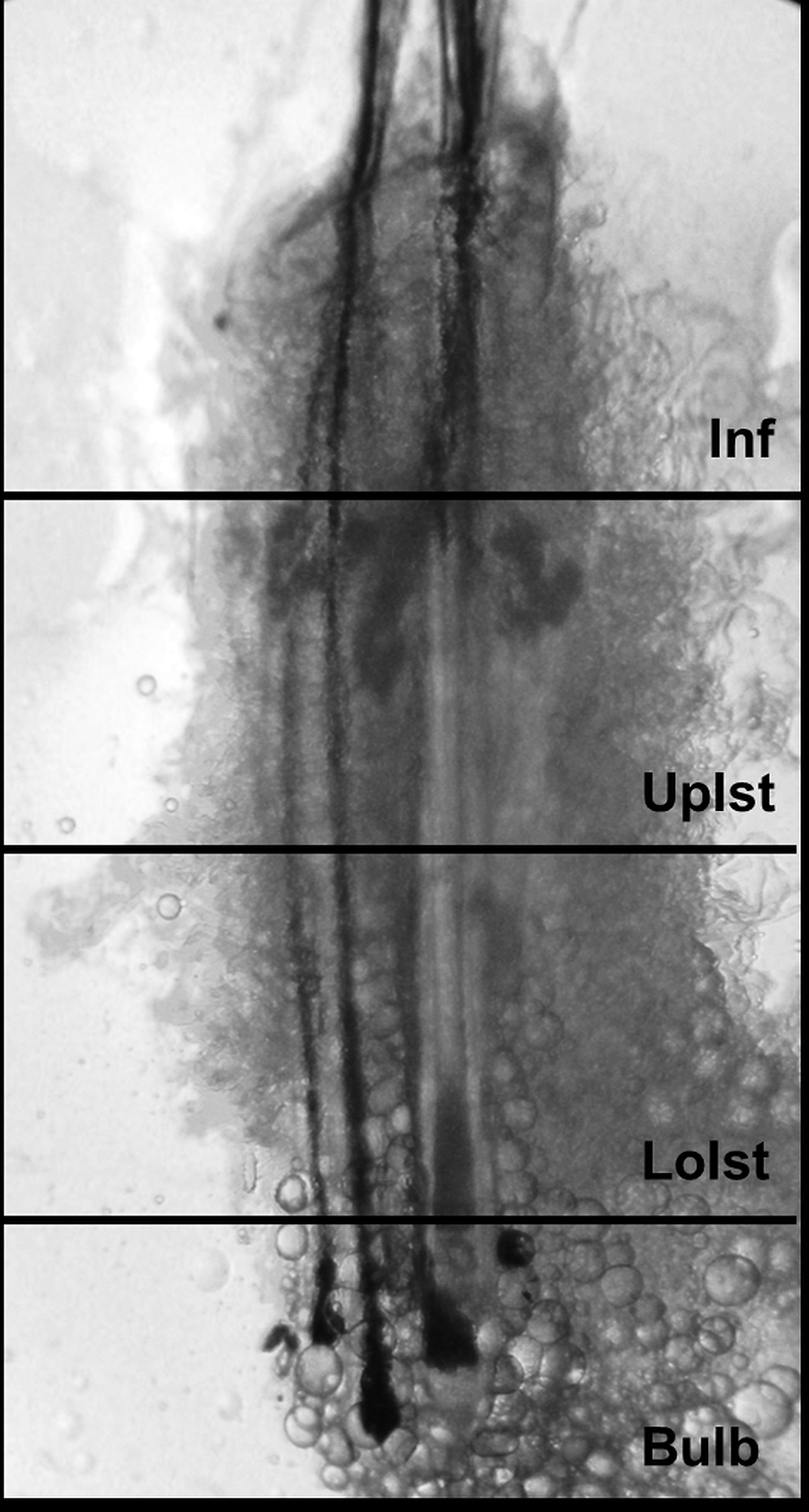

Supplement: S1 Fig — Hair follicles were microdissected in three fragments: Upper isthmus, lower isthmus and bulbar region. Inf, infundibulum; UpIst, upper isthmus; LoIst, lower isthmus; Bulb, bulbar region. (TIF) [file pone.0146937.s001.tif]

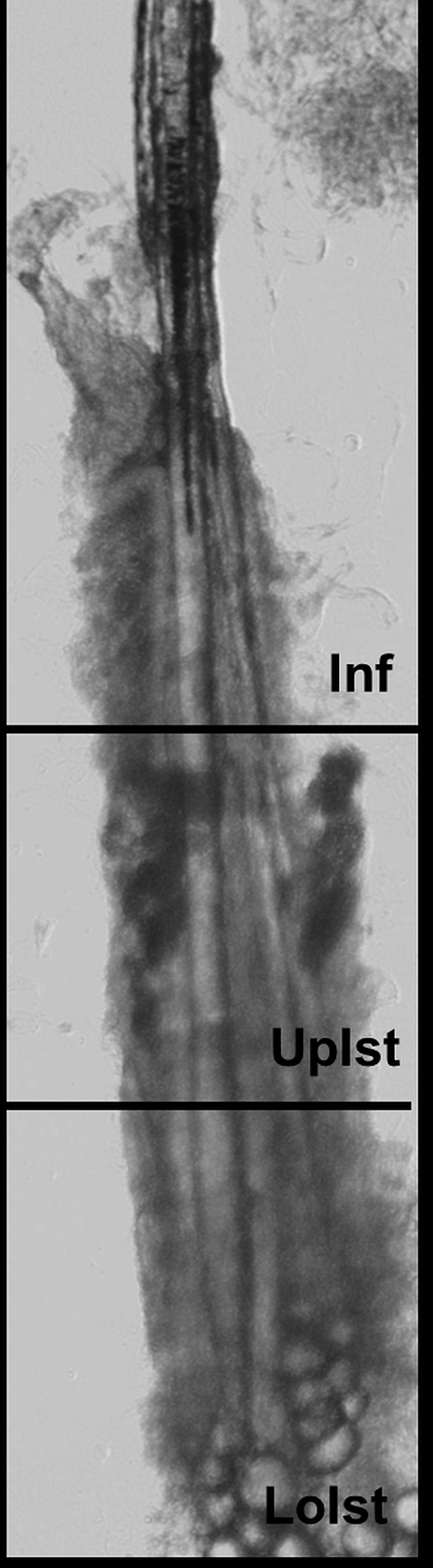

Supplement: S2 Fig — Hair follicles were microdissected in two fragments: Upper and lower isthmus. Inf, infundibulum; UpIst, upper isthmus; LoIst, lower isthmus. (TIF) [file pone.0146937.s002.tif]
